# An Algorithm Using Administrative Data to Identify Patient Attachment to a Family Physician

**DOI:** 10.1155/2015/967230

**Published:** 2015-08-27

**Authors:** Sylvie Provost, José Pérez, Raynald Pineault, Roxane Borgès Da Silva, Pierre Tousignant

**Affiliations:** ^1^Direction de Santé Publique du Centre Intégré Universitaire de Santé et Services Sociaux du Centre-Est-de-l'Île-de-Montréal, 1301 rue Sherbrooke E., Montréal, QC, Canada H2L 1M3; ^2^Centre de Recherche du Centre Hospitalier de l'Université de Montréal, 3480 rue Saint-Urbain, Hôtel-Dieu (Pavillon Masson), Montréal, QC, Canada H4W 1Y1; ^3^Institut de Recherche en Santé Publique de l'Université de Montréal, 7101 avenue du Parc, Montréal, QC, Canada H3N 1X9; ^4^Institut National de Santé Publique du Québec, 945 avenue Wolfe, Québec, QC, Canada G1V 5B3; ^5^Faculté des Sciences Infirmières, Université de Montréal (Pavillon Marguerite d'Youville), C.P. 6128, succursale Centre-ville, Montréal, QC, Canada H3C 3J7; ^6^Department of Epidemiology, Biostatistics and Occupational Health, McGill University, 1020 Avenue des Pins Ouest, Montreal, QC, Canada H3A 1A2

## Abstract

*Background.* Commonly self-reported questions in population health surveys, such as “do you have a family physician?”, represent one of the best-known sources of information about patients' attachment to family physicians. Is it possible to find a proxy for this information in administrative data? *Objective.* To identify the type of patient attachment to a family physician using administrative data. *Methods.* Using physician fee-for-service database and patients enrolment registries (Quebec, Canada, 2008–2010), we developed a step-by-step algorithm including three dimensions of the physician-patient relationship: patient enrolment with a physician, complete annual medical examinations (CME), and concentration of visits to a physician. *Results.* 68.1% of users were attached to a family physician; for 34.4% of them, attachment was defined by enrolment with a physician, for 31.5%, by CME without enrolment, and, for 34.1%, by concentration of visits to a physician without enrolment or CME. Eight types of patient attachment were described. *Conclusion.* When compared to findings with survey data, our measure comes out as a solid conceptual framework to identify patient attachment to a family physician in administrative databases. This measure could be of great value for physician/patient-based cohort development and impact assessment of different types of patient attachment on health services utilization.

## 1. Introduction

The medical literature has demonstrated that the patient-physician relationship represents primarily the interpersonal association of two parties bound by the nature of medical care. This bond, built on mutual cooperation, loyalty, and responsibility, is likely to influence the patient's use of health services and predict different health outcomes [1–9]. Patient attachment to a family physician is often understood as the relational dimension of the concept of continuity of care [10, 11]. It relies on a relationship developed through provision of therapeutic care and consecutive episodes of care taking place within the entire health system [6, 7, 12]. It is also rooted in a formal or informal contract between patients and their physicians and implies a sustained partnership and strong interpersonal relationship [13]. Thus, patient attachment to a family physician represents a main conceptual framework to analyze and interpret data on health services utilization. Identification of the patient's type of attachment to a family physician is of great interest for health population research initiatives, when used either as an explanatory or a confounding factor.

The patient-physician relationship can be framed from the physician or the patient perspective. While physicians can be surveyed to ask whether patients they have seen are their patients or not, the patient's perspective is usually assessed by health population surveys through self-reported questions such as “do you have a family physician?” or “who is your family physician?” Though they sound simple, these questions are designed to measure attachment to a family physician, a term that does not necessarily mean the same thing to all patients. Ideally the concept of “family doctor” refers to having a regular physician who provides primary care services while also following up and coordinating care for a patient [14–16], but a family doctor can also simply be, for many patients, the general practitioner they see most often. In addition, the patient-physician link may vary in intensity—ranging from patient's enrolment with a physician to occasional visits for acute problems that may occur once—a distinction which cannot be made with the question “do you have a family doctor?” Besides, this question rarely determines if the identified physician is a general practitioner or a specialist.

In administrative data, attachment to a family physician is often approximated by the usual provider of care (UPC) index [17], as the physician to whom a patient makes the majority of his or her visits has a greater probability to be this person's family physician. Attachment to a family physician can also be determined in administrative data by the enrolment of patients when available. A number of papers have looked at the identification of doctors' practice populations using administrative data to evaluate physicians' performance with their patients and to support provider-based analyses [18–27]. In the algorithm they used to identify patient attachment to a family doctor, Atlas et al. [24] included enrolment, physician type of practice (solo or collaborative), patient age, months since last visit to a physician, and distance between the patient's residence and the practice site. Katz et al. [22] used the criteria primary care visits to the same physician and cost of care (type of visits, referrals to specialists, laboratory tests, and imaging services) to assign patients to physicians. To assign regular providers of care to patients in administrative data, Shah et al. [27] found that an algorithm using the largest number of visits to a family physician had a good concordance with patients' identification of their regular family physician in survey data. However, to our knowledge, no algorithm has been developed to describe more precisely patient type of attachment to a family physician in administrative data.

### 1.1. Objective

The objective of this study is to establish, through a step-by-step algorithm based on administrative data, the attachment between patients and family physicians, as a proxy for the answers to the question “do you have a family physician?” or “who is your family physician?” The aim of our project is to propose a framework regarding types of patient-family physician relationship, rather than identifying with certainty the patients' family physician. To characterize types of patient-physician relationship, we focused on three primary care dimensions, for which information was available in our administrative databases: patient enrolment, complete medical examinations, and concentration of visits to one physician. This process oriented framework represents a first step in the development of a patient-level variable on attachment to a family physician that could be used in our analyses of health services utilization based on administrative data.

This study is a secondary analysis of a major project undertaken by the Population Health and Health Services Research Team at Montreal's Public Health Department to develop indicators based on health services utilization so as to monitor the impact of Quebec's primary care reforms on the users' health [28, 29].

### 1.2. Dimensions of Patient Attachment Used in the Algorithm

#### 1.2.1. Patient Enrolment

In Quebec, access to physicians is an open process through which patients can seek consultations with any family physician. Patient enrolment with a family physician associated with financial incentives was implemented to increase continuity of care and accessibility, as part of a reorganization renewal transformation of Quebec primary care medical services initiated in 2003. From the patient's perspective, being formally enrolled represents a reciprocal commitment, whereby the general practitioner becomes the patient's regular provider of care (except in case of an emergency) and the patient accepts this exclusive link with a physician. For this reason, we used the explicit nature of patient enrolment to confirm whether or not a patient had a family physician.

#### 1.2.2. Complete Medical Examination

In Quebec, a relatively large number of patients, most of them presenting minor health conditions, are not enrolled with a regular physician. However, many of these patients have a family physician. To include these patients in our definition of attachment to a family physician, we considered a complete annual medical examination (CME) claimed by a physician as an essential piece of information to assert an existing level of patient attachment. In most cases, the CME is carried out, usually on an annual basis, by the family physician the patient considers being the regular care provider. Such exams are strong determinants of receiving preventive services [30]. Physician's field experience suggests that provision of two CMEs to a patient in two consecutive years by the same physician is a strong indication that this physician is the patient's family physician. Even a single CME in a two-year period was judged relevant to determine a certain level of patient attachment.

#### 1.2.3. Concentration of Visits

For patients who are not enrolled and who have not had a complete CME in the past two years, we looked at usual provider of care to identify the general practitioner likely to play the role of the patient's family physician. Concentration of visits to the same doctor over time is often used as a proxy for relational continuity measured with data from administrative databases on service utilization. Prolonged or repeated contact with the same health care provider is presumed to result in stronger relationship, more effective use and sharing of information, and more consistent care management [10]. Although concentration of visits does not guarantee patient attachment to the physician seen most often, it probably indicates a certain level of patient-physician attachment. However, such an attachment may be weaker than one linked to enrolment or to having had one or more complete medical examinations.

## 2. Methods

### 2.1. Data Sources and Variables

In Quebec, medical services provided by family physicians are covered by the provincial health insurance plan (Régie de l'Assurance Maladie du Québec or RAMQ). Study data came from a 10-year database (physician fee-for-service database and patients enrolment registries), requested from the RAMQ to monitor the impact of Quebec's primary care reforms on users' health [28, 29]. A two-year data period was used for the construction of our algorithm. Due to the gradual implementation of patient enrolment since 2003, we used the physician fee-for-service database and patients enrolment registries for the most recent two-year data period at our disposal (financial years 2008-2009 and 2009-2010). The population under study (*n* = 1,248,249) comprised all patients aged 20 years and over, residents of Montreal, and active users of the health system, that is, patients with at least one ambulatory or hospitalization record within the two years of the study, excluding patients in long-term care facilities or deceased. Our study population represents 83.1% of the residents of Montreal aged 20 years and over.

The variables used to construct the algorithm were enrolment, complete medical examination, and UPC score. Two types of patient enrolment exist in Quebec. The primary care reform initiated in 2003 that included the creation of new organizational models, the family medicine groups (FMGs), aimed to improve accessibility and continuity related to a wide range of primary care, specialists, and social services [31, 32]. A typical FMG consists of 6 to 10 family physicians working with nurses to provide services for 8,000 to 15,000 registered patients. FMGs contract with the health ministry and agree to increase service provision (e.g., extended opening hours and 24/7 phone access) in exchange for complementary public funding for computerization and additional staff such as nurse. Family physicians working in FMGs can enroll their patients, regardless of their specific medical conditions. The reform has also included the possibility, for physicians in primary health care (PHC) clinics (FMGs, group or solo practices, community health centres, and family medicine teaching units), to enroll patients as vulnerable if they present specific conditions (see Appendix A for the list of eligible conditions) in order to provide them with more continuous and accessible healthcare services. FMG/vulnerable patient enrolment in ambulatory settings is formalized through a document signed by both the physician and the patient and entered into RAMQ patients enrolment registries, whether or not the physician is paid on a fee-for-service or a time basis (sessional fees for clinical activities). The registries contain, for each patient on an annual basis, information regarding the dates at which enrolment begins and ends. In order to identify a sustained link between patients and family physicians, enrolment for at least 18 months in the two-year period of the study was used as criterion for identification of attachment.

For the identification of complete medical examination and the calculation of UPC score, visits to family physicians in PHC clinics were identified through the RAMQ physician fee-for-service database since the vast majority of Quebec family physicians in PHC clinics are fee-for-service paid. In contrast, physicians working in community health centres or family medicine teaching units are mostly paid on a time basis; consequently, they were excluded from the calculation of the UPC score or the identification of CMEs in the database. However, since enrolment of patients in those settings is usually the rule and could be identified in the RAMQ patients enrolment registries even if the physician is paid on a time basis, those physicians were included in the identification of patient-physician relationship through enrolment. No further exclusions were applied. Regarding CME, a Quebec physician cannot bill for this examination more than once a year for a patient in a PHC clinic (see Appendix B). The UPC score was calculated, for patients with two or more visits in a PHC clinic in the two-year study period, as the number of patient visits (excluding visits to emergency rooms or during hospitalization) to the most frequently seen family physician in a PHC clinic over the total number of patient visits to all family physicians in PHC clinics during the period. As we assumed that repeated contacts with a family physician indicate a sustained patient-physician relationship, we interpreted conservative UPC scores of 75% or higher as a significant aspect of patient attachment.

The algorithm applied a hierarchical order of PHC dimensions (enrolment, CME, and concentration of visits) that are likely to bind physicians and their patients from seemingly strong (enrolment) to weak (a single patient visit in a PHC clinic without enrolment) levels of attachment. During the classification process, we assigned only one attachment type to each patient and concurrently identified the associated physician. Patient and physician data were identified and linked through unique encrypted ID numbers.

### 2.2. Steps of the Algorithm

#### 2.2.1. Identifying Attachment to a Family Physician through Patient Enrolment: Steps 1 and 2

**Figure 1:**
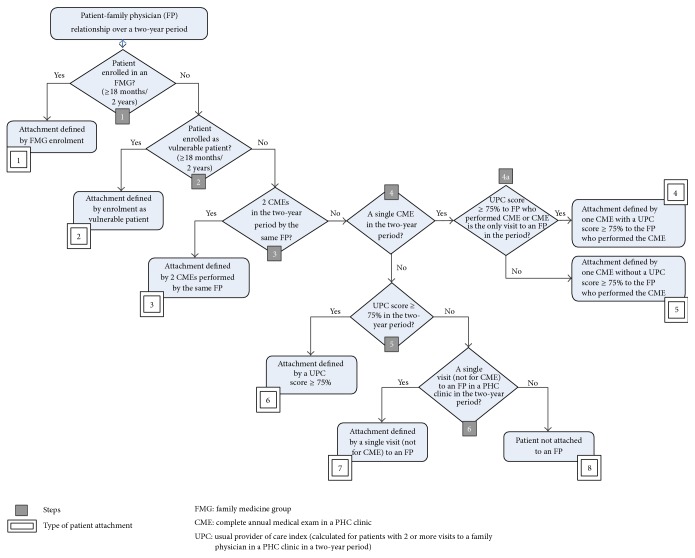
Stepwise classification scheme to designate the type of patient attachment to a family physician (over a two-year period).

Enrolment, the algorithm first dimension, is defined in steps 1 and 2 of Figure 1: first by FMG enrolment for at least 18 months in the two-year study period and then, for the patients not enrolled in an FMG, by enrolment as vulnerable patient for at least 18 months. If a patient was identified as having both enrolments, FMG enrolment was prioritized. In this manner, types of attachment 1 and 2 were created.

#### 2.2.2. Identifying Attachment to a Family Physician through CME: Steps 3 and 4

The second dimension is the presence of a complete annual medical examination (CME) performed by a family physician in a PHC clinic. For patients without any type of enrolment, in step 3 (Figure 1), we looked for two CMEs claimed by the same family physician in the two-year study period. Therefore, patients who met this criterion were assigned to type 3 of attachment. In steps 4 and 4a, patients with only one CME for the same period of time were included. Among these patients, we distinguished those (type 4) with a concentration of visits (UPC score of 75% or higher) to the physician having performed the CME from those (type 5) without such a concentration of visits to the physician having performed the CME. Patients with more than one CME performed by different physicians did not meet the criteria for either of types 3 to 5.

#### 2.2.3. Identifying Attachment to a Family Physician through Concentration of Visits: Steps 5 and 6

The third dimension of our algorithm is UPC score calculated for each patient over a two-year period. At step 5 (Figure 1), among patients not enrolled and who did not have a CME in the two-year study period, we classified patients with a UPC score 75% or higher as type 6. At step 6, type 7 designated nonenrolled patients with a single visit (not a CME) to a family physician in a PHC clinic in the two-year period. Finally, type 8 included all patients without a designated attachment type.

In summary, our 8-type measure of patient attachment to a family physician over a two-year period reads as follows:

1. attachment defined by FMG enrolment;
2. attachment defined by enrolment as vulnerable patient, without FMG enrolment;
3. attachment defined by two CMEs in a PHC clinic by the same physician, without enrolment;
4. attachment defined by one CME in a PHC clinic with a UPC score of 75% or higher to the physician who performed the CME, without enrolment;
5. attachment defined by one CME, without enrolment or UPC score of 75% or higher to the physician who performed the CME;
6. attachment defined by a UPC score of 75% or higher in a PHC clinic, without enrolment or CME;
7. attachment defined by a single patient visit (not for CME) to a family physician in a PHC clinic, without enrolment;
8. patient not attached to a family physician.

## 3. Results

**Figure 2:**
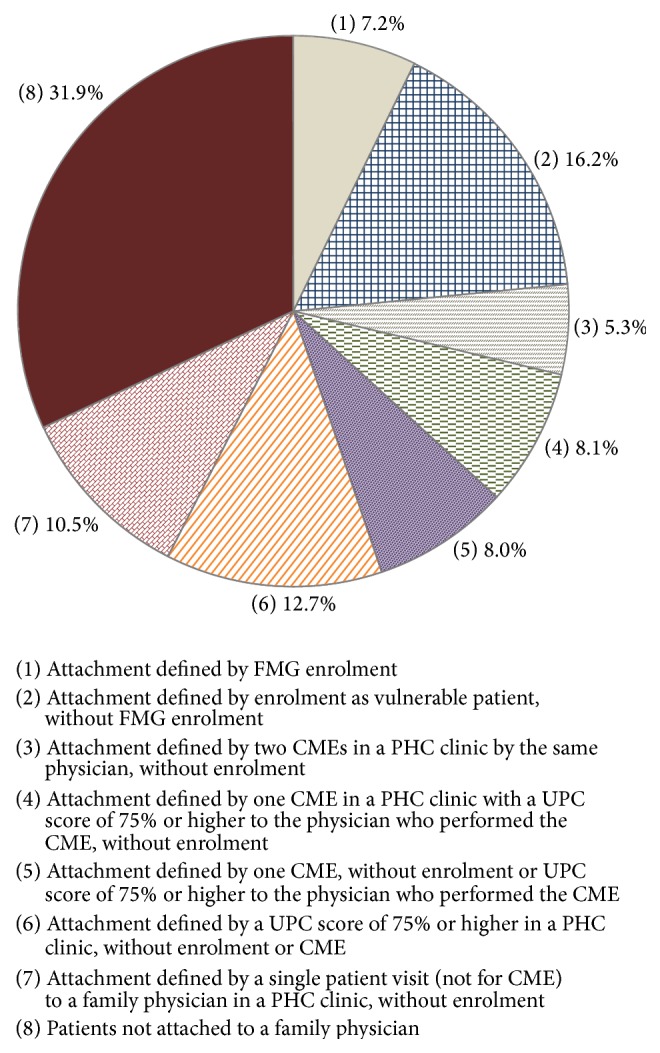
Distribution of the study population by type of patient attachment to a family physician over a two-year period. Health services users aged 20 years and over (*n* = 1,248,249), 2008–2010.

In all, 68.1% of users were attached to a family physician (types 1–7) in a two-year period (2008-2009 and 2009-2010).  Figure 2 presents the study population distribution (*n* = 1,248,249) for each type of patient attachment.

Results suggest that type 8 patients could also be described as inconsistently attached to a particular family physician. More specifically, 20.4% of all users of health services had more than one visit to a family physician over the two-year period but were classified as nonattached, given that no formal type of enrolment or complete medical examination was found, and no UPC scores of 75% or over were detected (Table 1). Although medical care was indeed provided to these patients through multiple encounters with different family physicians, we were unable to determine patient attachment to a family physician under such circumstances. By adding the five categories of unattached patients listed in Table 1, we can extend our measure of attachment to a 12-type measure to support detailed analyses of patient attachment [33].

As a result of evaluating eligibility to attachment types, patients were assigned to one type only. Yet they could have more than one patient-physician relationship characteristic or dimension. We completed our descriptive portrait by calculating the prevalence of the three main dimensions of our algorithm (enrolment, CME, and concentration of visits) for each type of patient attachment (Table 2). For example, by the time patients enrolled in an FMG formed type 1, 34.8% of them were also enrolled as vulnerable patients, 14.9% had two CME, and 39.4% had a minimum UPC score of 75%.

Prevalence of each dimension of attachment in health services users is presented in Table 3. It shows the percentage of users who would have been identified as attached to a family physician, if each dimension of attachment was considered independently of others. For example, for the two-year study period, looking at enrolment as vulnerable patients alone would have allowed us to identify attachment for 18.7% of users.

Table 4 compares some sociodemographic and health data in the 8 groups of patients identified by the algorithm. The proportion of unattached patients and of patients for whom the attachment was defined only by a single visit (not for CME) to a family physician in a PHC clinic, without enrolment, was higher among males, younger individuals, and patients with a lower level of morbidity. The proportion of unenrolled patients for whom the attachment to a family physician was defined through the presence of two CMEs by the same physician in the two-year period was higher in females than in males. Attachment through enrolment as vulnerable patient was more frequent in sicker and older patients.

## 4. Discussion

Our figure of 68.1% of Montrealers (aged 20 years and over) attached to a family physician is comparable to those obtained through population surveys. The Canadian Community Health Survey estimated that 67.4% of Montrealers (12 and over) had a family physician in 2010 [34], while the EQES (Enquête Québécoise sur l'Expérience de Soins) developed in 2010-2011 by the Institut de la Statistique du Québec revealed that 68.8% of Montrealers (15 and over) had a family physician [35]. Moreover, another population survey conducted in 2010 to assess the evolution of primary healthcare organizations showed that 66.4% of Montrealers (20 and over) reported having a family physician [36].  Appendix C presents some characteristics of patients with and without a family physician issued from that survey carried out in the same population [36]. As shown by the results of our algorithm using administrative data, the proportion of unattached patients in the survey data was higher among males and among younger subjects. The proportion of unattached patients among diabetic patients in that survey was also quite similar to the proportion we observed in the administrative data.

These comparisons suggest that identification of patients attachment to family physicians through our algorithm may be an interesting proxy, in administrative data, for the answers to the question “do you have a family physician?” or “who is your family physician?” However, a physician seen once over a two-year period, without enrolment or CME, may not be the physician the patient would identify as his or her family doctor. Conversely, a patient could have a family physician without having seen him or her during the two-year study period. Our current data did not allow us to reliably validate our identification of each patient's family physician in administrative data. Such validation would have required linking our administrative patients data to patient survey data or to physician data on identification of their patients [24, 27], which could not be done with our data.

Even if the variable we constructed did not assure us that the physician to which a patient is attached, according to our algorithm, is effectively the one considered to be the patient's family physician, it nonetheless allowed us to characterize, in administrative data, the type of attachment of the patient to a family physician over the study period. Since the aim of our project was to propose a framework for defining types of patient-family physician relationship rather than identifying with certainty the patient's family physician, we think that our approach is valuable. Moreover, creating an attachment variable that includes different categories or types of attachment enhances the precision in analysis of attachment itself, with regard to patient characteristics related to different categories of attachment as well as to the impact of different types of attachment on various outcomes. In that regard, our results indicate that patient characteristics vary among types of attachment. For example, the proportion of attachment defined by the presence of two CMEs by the same physician in the two-year period was higher in females, which may reflect a profile of health services utilization associated with preventive services as Pap test or breast examination. Our results also show that although sicker and older patients appeared to have a more important attachment to family physicians (notably through enrolment), a nonnegligible proportion of those patients were unattached to a family physician during the study period.

Contrary to the algorithms developed by others [18–20, 23, 24], our objective was not to identify physicians' practice populations, but rather to create a variable that describes each patient's attachment to a family physician. Although closely linked to the availability of administrative data, our choice of attachment dimensions to construct our algorithm aimed mostly to reflect certain aspects of the patient-family physician relationship: formal enrolment, type of examination reflecting care comprehensiveness, and concentration of visits. Enrolment is quite specific: a patient cannot be enrolled in an FMG or as vulnerable without having a family physician. Hence, the first step of our algorithm relates to enrolment, which clearly indicates that the patient has a family physician. For patients who were not enrolled, we used information available in administrative data likely to reveal a link between them and a family physician. This is why we included, for patients who were not enrolled, information on complete annual examination and concentration of visits to characterize the patient-family physician relationship. It should be noted that although the pertinence of a complete annual examination is currently reconsidered, it was still considered as a usual procedure in family practice during the study period.

Using these dimensions of the patient-family physician relationship allowed us to increase the sensitivity of our algorithm. For the two years considered in this study, attachment to a family physician for nearly 45% additional patients could be identified by adding to enrolment alone, concentration of visits (including patients with only one visit), and provision of at least one complete medical examination. It is interesting to note that about 40% of enrolled patients had at least one complete medical examination during the two-year study period. In addition, a majority of patients who were not enrolled in an FMG but only as vulnerable had high concentrations of visits with the enrolling physician (64.8%). Similar results were observed regarding concentration of visits to the same physician for patients who had two complete medical examinations. The figure is lower for patients enrolled in FMGs (39.4%), probably due to the nature of this organizational model that fosters group practice. Moreover, the higher proportion of patients who saw a family physician only once among those enrolled in FMGs compared with those enrolled as vulnerable without FMG enrolment probably reflects complementary follow-up by multidisciplinary teams in FMG (especially nurses), which is not readily detectable in administrative data.

### 4.1. Limits

In addition to the issue of validation that has been already discussed, our study has some limits. The construction of our algorithm depends largely on the availability of relevant and accurate information in administrative data to characterize the patient-family physician relationship. Complementary data, such as type of PHC practice of the family physician, would have been useful but were not available.

The construction of our variable is based on a two-year period. One objective of our algorithm was to create, in our database, a variable characterizing the patient-physician relationship that could eventually be used in our analyses of health services utilization covering one-year and two-year periods. Since the type of patient-physician relationship may vary over time according to patient age or health status, we chose to measure this relationship over a relatively short period of time. However, we chose a period sufficiently long to capture two consecutive annual visits, concentration of visits to a physician, and significant enrolment.

Finally, although our algorithm depends on specific information available in administrative data in Quebec, a similar approach could be used with data available in other contexts regarding similar dimensions characterizing patient-family physician relationship, namely, enrolment, certain types of exams, and concentration of visits to the same physician. We tend to see the proposed algorithm more as a conceptual framework that can be adapted to different contexts and queries than a single and unique way to characterize the link between patients and family physicians. For example, analysis of the intensity or degree of the patient attachment through additional configurations or combinations of the dimensions used in our algorithm could help identify specific elements of the patient attachment to a family physician more likely to be associated with various outcomes. It should be noted that results relating to outcomes of care are beyond the scope of the present paper.

## 5. Conclusion

Our study aimed to present an algorithm to characterize the attachment between patients and family physicians in administrative databases. Compared to identification of the regular family physician that can be obtained from patients' surveys, our 8-type measure of patient attachment generates a more refined description of the phenomena underpinning patient attachment to a family physician. This measure could be of great value for impact assessment of patient attachment on health services utilization using administrative data. By describing patients' attachment to family physicians through different categories, our measure provides the opportunity to assess the impact of patient attachment on different outcomes and more specifically on chronic disease management and preventive services delivery in a more precise way than by just knowing whether or not the patient has a family physician. Hence, our measure of attachment represents an interesting tool in order to better understand the way patients' attachment to family physicians is associated with health services utilization. The identification of the patient's type of attachment to a family physician could also be of great interest as a confounding factor in health services utilization research initiatives using administrative data. Finally, our algorithm allows us to identify the patient's family physician in our data. This identification enables us to pursue further data linkage and build physician/patient-based cohorts that can be followed through the continuum of care.

## Figures and Tables

**Table 1:** Characteristics of nonattached patients in a two-year period, among overall population. Health services users aged 20 years and over (*n* = 1,248,249), 2008–2010.

Patients enrolled by different physicians in an FMG or as vulnerable patients	0.4%

Patients without any type of enrolment with more than one CME performed by different physicians	1.0%

Patients without any type of enrolment or CME, but with a UPC score between 51% and 74%	4.6%

Patients without any type of enrolment, CME, or UPC score higher than 50%, but with more than one visit to family physicians in PHC clinics	14.4%

Patients without any type of enrolment or ambulatory visits to a family physician in a PHC clinic	11.5%

Total	31.9%

**Table 2:** Types and primary care dimensions of attachment to a family physician (FP). Health services users aged 20 years and over (*n* = 1,248,249), 2008–2010.

Primary care dimensions of attachment (over a two-year period)	Types of patient attachment		
	(over a two-year period)		
	Attachment defined by enrolment		Attachment defined by two CMEs done by the same FP, without enrolment
	To an FMG physician	As vulnerable patient, without FMG enrolment	
	*n* = 90,264	*n* = 202,335	*n* = 65,732
	%	%	%
FMG enrolment (≥18 months)	100	—	—

Enrolment as vulnerable patient (≥18 months)	34.8	100	—

2 CMEs	14.9^*∗*^	17.6^*∗*^	100

1 CME	25.7^*∗*^	23.6^*∗*^	—

UPC ≥ 75% (patients with ≥ 2 visits to an FP in a PHC clinic)	39.4^*∗*^	64.8^*∗*^	65.5

A single patient visit (not CME) in a PHC clinic	9.9^*∗*^	3.8^*∗*^	—

^∗^May be slightly underestimated since UPC score calculation and CME identification could not be done for patients enrolled in community health centers or family medicine teaching units, where FP are mostly paid on a time basis.

**Table 3:** Prevalence of each dimension used to build the attachment variable in the two-year period. Health services users aged 20 years and over (*n* = 1,248,249), 2008–2010.

FMG enrolment (≥18 months)	7.2%

Enrolment as vulnerable patient (≥18 months)	18.7%

2 CMEs performed by the same family physician	9.2%

A single CME performed	21.8%

UPC score ≥ 75% to a family physician (calculated for patients with 2 or more visits to a family physician in a PHC clinic)	35.7%

A single patient visit (not CME) in a PHC clinic	14.1%

**Table 4:** Characteristics of patients according to their type of attachment to a family physician. Health services users aged 20 years and over (*n* = 1,248,249), 2008–2010.

Types of patient attachment (over a two-year period)	All patients	Sex		Age group				Chronic diseases	Morbidity level^†^		
		Female	Male	20–44	45–59	60–74	75 or over	Diabetes	Low	Moderate	High
		(*n* = 687,680)	(*n* = 560,569)	(*n* = 547,257)	(*n* = 343,368)	(*n* = 225,610)	(*n* = 132,014)	(*n* = 116,657)	(*n* = 461,851)	(*n* = 513,623)	(*n* = 111,324)
	%	%	%	%	%	%	%	%	%	%	%
FMG enrolment	7.2	8.0	6.2	6.3	8.1	7.9	7.7	8.1	6.8	7.8	8.0
Enrolment as vulnerable patient (but not FMG)	16.2	17.7	14.4	2.9	9.5	26.7	71.2	48.4	7.4	23.5	36.9
2 CMEs by the same FP, without enrolment	5.3	6.2	4.2	3.7	7.7	8.0	0.7	2.8	6.1	6.6	2.9
1 CME with a UPC score ≥75% to the CME FP, without enrolment	8.1	7.9	8.3	7.7	10.7	8.8	1.6	4.5	9.5	7.4	4.4
1 CME, without enrolment or UPC score ≥75% to the CME FP	8.0	9.2	6.5	10.1	8.9	5.7	1.1	3.6	8.8	9.1	6.2
UPC score ≥75%, without enrolment or CME	12.7	12.3	13.2	11.1	15.6	16.6	5.2	11.8	13.0	13.6	11.0
A single visit (not for CME) to an FP, without enrolment	10.5	8.4	13.2	15.8	9.0	5.0	2.4	3.4	13.0	4.5	4.9

Patients attached to an FP	68.0	69.7	66.0	57.6	69.5	78.7	89.9	82.6	64.6	72.5	74.3

Patients not attached to an FP	31.9	30.3	33.9	42.5	30.4	21.3	10.1	17.5	35.6	27.5	25.7

Total	100%	100%	100%	100%	100%	100%	100%	100%	100%	100%	100%

^†^Morbidity level is defined by the RUB (Resource Utilization Band, ACG Case-Mix System), which identifies 5 categories of morbidity with similar expected health services utilization (1: healthy patients; 2: low level of utilization; 3: moderate level of utilization; 4: high level of utilization; and 5: very high level of utilization). Low level of morbidity corresponds to RUB 1 and 2; moderate level of morbidity to RUB 3; and high level of morbidity to RUB 4 and 5.

**Table 5:** (Appendix C) Characteristics of patients having a family physician (FP) or not. Respondents aged 20 years and over (*n* = 5140), Montreal, 2008–2010. Results from the population survey of the project Assessing the Evolution of Primary Healthcare Organizations and their Performance (2005–2010) in Two Regions of Québec Province: Montréal and Montérégie [36].

	All respondents	Sex		Age group				Chronic diseases
		Female	Male	20–44	45–59	60–74	75 or over	Diabetes
		(*n* = 2665)	(*n* = 2475)	(*n* = 2452)	(*n* = 1349)	(*n* = 834)	(*n* = 505)	(*n* = 390)
	%	%	%	%	%	%	%	%
Respondents who declared that they have an FP	66.4	72.6	59.6	51.5	72.6	86.2	89.3	82.8

Respondents who declared that they do not have an FP	33.6	27.4	40.4	48.5	27.4	13.8	10.7	17.2

Total	100%	100%	100%	100%	100%	100%	100%	100%

## Appendices

### A. Conditions Eligible to Enroll Patients as Vulnerable

The following conditions (list in use since 2008) determine if a patient can be enrolled as vulnerable:

- being aged 70 or over;
- mental disorders (DSM-IV): psychotic disorders, bipolar disorders, panic disorders, generalized anxiety disorders, pervasive developmental disorders (autism, Asperger), and eating disorders (anorexia, bulimia);
- active major depressive disorders, first episode;
- recurrent major depressive disorders;
- chronic obstructive pulmonary diseases (COPD), moderate to severe asthma (patient with FEV1 below 70% of the predicted value), and occupational lung diseases;
- arteriosclerotic heart disease, heart failure;
- cancer associated with prior, current, or projected systemic chemotherapy treatment, radiation treatment, or being in palliative phase;
- diabetes excluding gestational diabetes;
- drug addiction or alcoholism in withdrawal phase or having led to withdrawal treatment for hard drugs or alcohol during the past five years and methadone treatment for drug addiction;
- HIV/AIDS;
- degenerative central nervous system diseases;
- chronic inflammatory diseases: rheumatoid arthritis, psoriasis involving sites other than skin, lupus, scleroderma, and other collagen diseases, ulcerative colitis, and Crohn's disease;
- chronic kidney failure with creatinine clearance below 50 mL per minute;
- recurrent thrombosis requiring lifelong anticoagulation therapy and INR testing;
- atrial fibrillation requiring lifetime anticoagulation therapy and INR testing.

Source: RAMQ. Brochure no 1 - Omnipraticiens EP - MÉDECINE FAMILLE/PRISE EN CHARGE/SUIVI DE LA CLIENTÈLE. http://www.ramq.gouv.qc.ca/SiteCollectionDocuments/professionnels/manuels/104-brochure-1-omnipraticiens/000_complet_entente_omni.pdf (Accessed April 29, 2015).

### B. Complete Annual Medical Examination

A description of a complete annual medical examination is included in the RAMQ billing manual. The examination includes the following elements:

1. a questionnaire on (a) the patient's family history; (b) the patient's personal history; (c) the medical reason for physician use; and (d) how the following structures and systems are working: ear, nose, and throat; eyes; digestive system; cardiovascular system; respiratory system; genitourinary system; nervous system; musculoskeletal system; and endocrine system;
2. clinical examination of the following: skin and appendages of the skin; lymph nodes; head; neck; thorax; abdomen; genitals, unless contraindicated; and spinal column and extremities;
3. recommendations for the patient;
4. recording in the patient's medical record any data the physician deems to be significant.

A physician cannot bill for this examination more than once a year for each patient in a PHC clinic. The duration of this examination usually takes about 45 minutes.

Source: RAMQ. Manuel des médecins omnipraticiens (no 100)/Préambule général/Examens. http://www.ramq.gouv.qc.ca/fr/professionnels/medecins-omnipraticiens/manuels/pages/facturation.aspx (Accessed August 23, 2015).

### C. Characteristics of Patients Having a Family Physician or Not: Results from a Population Survey, Montreal, 2010 [36]

See Table 5.
